# Unsupervised learning for large-scale corneal topography clustering

**DOI:** 10.1038/s41598-020-73902-7

**Published:** 2020-10-12

**Authors:** Pierre Zéboulon, Guillaume Debellemanière, Damien Gatinel

**Affiliations:** 1 Department of Ophthalmology, Rothschild Foundation, 25 Rue Manin, 75019 Paris, France; 2 CEROC (Center of Expertise and Research in Optics for Clinicians), Paris, France

**Keywords:** Computational science, Corneal diseases

## Abstract

Machine learning algorithms have recently shown their precision and potential in many different use cases and fields of medicine. Most of the algorithms used are supervised and need a large quantity of labeled data to achieve high accuracy. Also, most applications of machine learning in medicine are attempts to mimic or exceed human diagnostic capabilities but little work has been done to show the power of these algorithms to help collect and pre-process a large amount of data. In this study we show how unsupervised learning can extract and sort usable data from large unlabeled datasets with minimal human intervention. Our digital examination tools used in clinical practice store such databases and are largely under-exploited. We applied unsupervised algorithms to corneal topography examinations which remains the gold standard test for diagnosis and follow-up of many corneal diseases and refractive surgery screening. We could extract 7019 usable examinations which were automatically sorted in 3 common diagnoses (Normal, Keratoconus and History of Refractive Surgery) from an unlabeled database with an overall accuracy of 96.5%. Similar methods could be used on any form of digital examination database and greatly speed up the data collection process and yield to the elaboration of stronger supervised models.

## Introduction

Machine learning (ML) is getting more and more traction in many fields of medicine^1^ including ophthalmology^2^. ML has been used successfully in a wide range of applications in ophthalmology including photograph based diabetic retinopathy detection^3^, Optical Coherence Tomography (OCT) diagnosis^4^, image segmentation^5^, intraocular lens calculation^6,7^ and refractive surgery screening^8^. Most ML algorithms are supervised and therefore require a large quantity of manually labeled data. All of our digital diagnostic tools (topography, OCT, optical biometers etc.…) have enormous databases of examinations that could be used for research purposes but are usually unlabeled and hence unusable. The manual labeling process is very time-consuming for physicians and it would be interesting to be able to automatically label or pre-label these large amounts of data to constitute groups for clinical studies. These large groups of labeled examinations could for example help improve screening and diagnostic tests or help to understand disease pathophysiology using the power of data science and machine learning. Unsupervised learning^9^ is the field of ML that uses unlabeled data and specifically, clustering is its most common application. It uses algorithms that have no prior knowledge of the data labels to regroup data in clusters based on data similarity. In the field of ophthalmology, recent studies have shown that it is possible to effectively perform clustering for keratoconus staging^10^ and glaucoma visual field^11^ and retinal nerve fiber layer^12^ pattern analysis. Nevertheless, little work has been done to highlight the capabilities of unsupervised learning to help collect and sort large amounts of data for use in subsequent studies. Corneal topography is the primary examination performed for refractive surgery screening, corneal diseases diagnosis and follow-up and contact lens fitting. Its computer-assisted version as we know it today has been used worldwide for at least thirty years^13^. Modern topographs capture precise curvature, elevation and thickness data of the cornea and present the results as color maps. In this study, we test the efficiency of unsupervised algorithms to extract and sort usable examinations from a large unlabeled corneal topography database into different diagnostic clusters, with little human intervention, data cleaning or feature selection.

## Results

**Figure 1:**
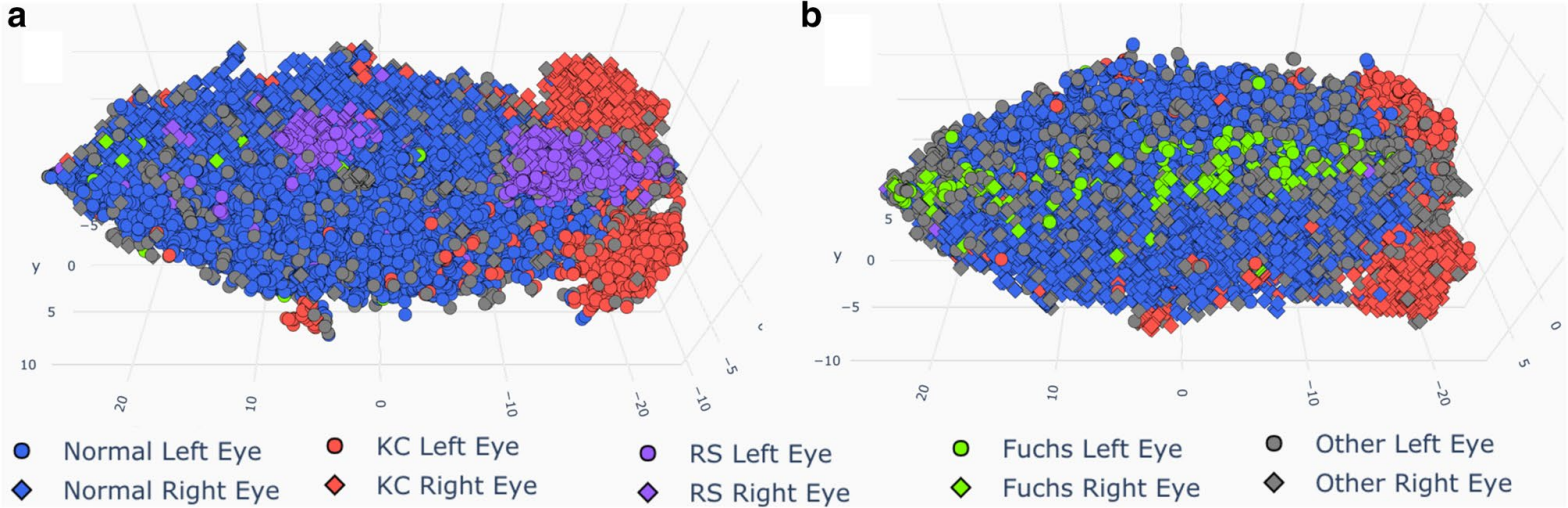
3 Dimensional representation of the dataset after dimensionality reduction. (**a**: Top view, **b**: Bottom view). Ground truth labels are color coded. Left and right eyes are represented respectively as squares and circles. (KC = Keratoconus, RS = History of Refractive Surgery, Fuchs = Fuchs endothelial dystrophy).

The repartition of all examinations’ ground truth diagnoses is shown in Table 1. The dataset consisted of 13,705 examinations, 6882 right eyes and 6823 left eyes of 6979 different patients. A three dimensional representation of the dataset, obtained from dimensionality reduction, is shown in Fig. 1 (and Supplementary Video 1) with ground truth diagnoses color-coded (two dimensional representations are available in Supplementary Figure S1).

**Table 1:** Ground truth diagnosis repartition in examinations included and excluded by the clustering algorithm.

Ground truth label	Examinations included (n = 7019)	Examinations excluded (n = 6686)	Total examinations (n = 13,705)
Normal	5775	4443	10,218
KC	796	521	1317
RS	325	353	678
Fuchs	29	206	235
Other	94	1163	1257

## Clustering results

**Figure 2:**
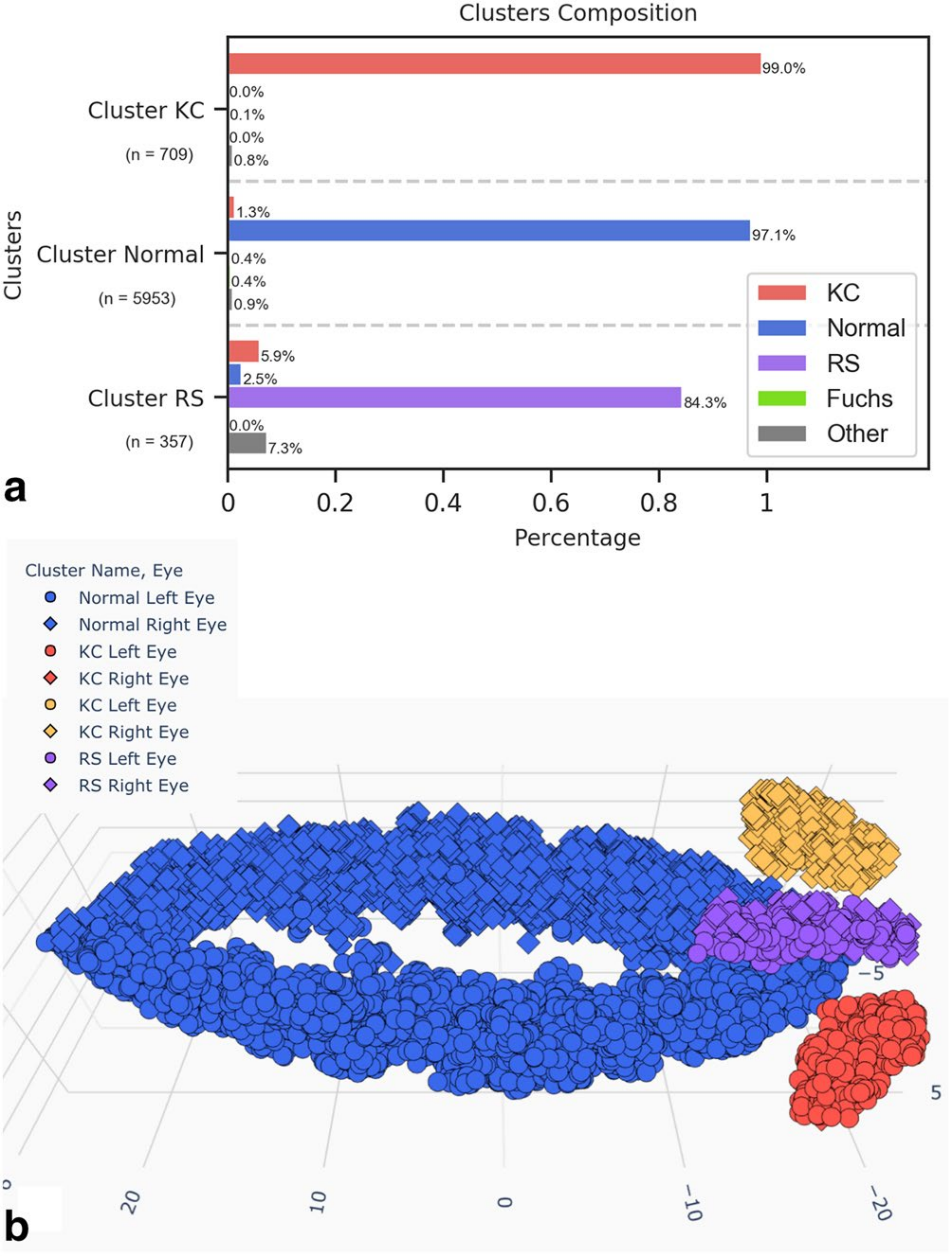
Clustering algorithm results. Clusters composition analysis (**a**), 3D representation of the results colored by cluster label (**b**). Each constituted cluster is represented by a different color. Red and yellow clusters both represent keratoconus clusters and are considered as one for the cluster composition analysis. (KC = Keratoconus, RS = History of Refractive Surgery, Fuchs = Fuchs endothelial dystrophy).

7019 examinations were included and clustered and 6686 examinations were excluded by the clustering algorithm. The ground truth diagnosis repartition of excluded and included examinations is shown in Table 1. Most examinations manually labeled as ‘Other’ were excluded by the clustering algorithm. The clustered data was organised in 4 clusters. These clusters represented Normal examinations, History of Myopic Refractive Surgery (RS) and Keratoconus (KC) diagnoses. The keratoconus examinations were separated into two clusters, one for the left eyes and one for the right eyes. For clarity purposes we will consider both keratoconus clusters together as one ‘KC’ cluster. Figure 2 shows the clusters resulting from the clustering algorithm and the clusters ground truth diagnosis composition. The same results colored by ground truth diagnosis are available in Supplementary Figure S2 and Supplementary Video 2.

The overall classification accuracy for the clustered examinations was 96.5%. Sensitivity and specificity for each diagnosis are reported in Table 2.

**Table 2:** Sensitivity (Sn) and specificity (Sp) for each diagnosis.

	Clustering algorithm results (n = 7019)	
	Sn	Sp
Normal	99.8%	85.9%
KC	88.0%	99.9%
RS	93.2%	99.2%

The mean ± standard deviation number of missing values per examination was higher for incorrectly classified examinations 349.4 ± 538.9 than for correctly classified examinations 52.4 ± 145.0 (*p* < 0.0001) and also higher for excluded examinations 128.6 ± 313.7 than for included examinations 65.3 ± 179.4 (*p* < 0.0001).

The number of correctly classified examinations with less than 1% and more than 1% of missing values were 3191 and 906 respectively, and 81 and 122 for incorrectly classified examinations. Chi Square independence test showed an association between the number of missing values and the number of correctly or incorrectly classified examinations (*p* < 0.0001).

## Discussion

This clustering method was efficient in both eliminating low quality examinations (with missing values and artefacts) and constituting relatively pure clusters of the most represented classes in the dataset with little human intervention. Even though roughly half of the dataset was excluded in the clustering process, the specificity and sensitivity results on the clustered examinations highlight our method’s performance in automatically extracting and sorting a large number of examinations from a noisy dataset.

Many algorithms have been described^14–17^ to perform diagnosis or screening on corneal topography and elevation data. Although our approach is not intended for precise diagnosis, it is interesting to note that, compared to previous methods, our technique is not based on human defined features. Indeed most other algorithms use specific parameters that were designed or selected by humans (for example I-S index or KISA). In our approach, on the other hand, we tried to use almost no human intervention in the feature selection process. The dimensionality reduction technique automatically created three features (x, y, and z axes of the 3D representation) from the raw data of 4 corneal maps frequently used by physicians in clinical practice. Those three features allowed the separation of corneal examinations in groups that are coherent to clinical diagnosis with no human intervention regarding which corneal parameter to use or not use.

Unsupervised learning has been previously used on corneal parameters in three studies.

The first one studied keratoconus severity with a somewhat similar methodology^10^. It showed that it was possible to separate examinations in groups of different severity according to the Ectasia Status Index (ESI) provided by an OCT based corneal instrument. The study population differed from ours as it was composed solely of examinations with a valid ESI measurement, whereas we used a large sample of unlabeled data with any kind of diagnosis and no exclusion criteria. Also, they selected 420 parameters for clustering analysis which were not detailed, whereas we used raw matrices of the 4 most frequently used topography maps in clinical practice. Our approach was to test topography clustering in a broader context in an attempt to roughly and automatically sort a large amount of data with very little a priori human feature selection or data cleaning. When considering only the normal and advanced keratoconus clusters, the authors report a specificity for diagnosis of normal cases of 94.1% and a sensitivity for keratoconus diagnosis of 97.7%. In our study, when considering only the normal and keratoconus clusters, those values would respectively be 100% and 81.2%. The results are not exactly comparable, as we considered all keratoconus and normal patients and did not exclude less severe cases that are more prone to be miss-classified as normal. The second study used Zernike polynomial decomposition of the corneal surface to perform unsupervised clustering of normal corneal anterior surfaces^18^. This study first showed that Zernike decomposition was an effective way to perform feature selection and dimensionality reduction for the anterior surface of the cornea. Second, the authors found that anterior surfaces of normal corneas could be clustered in 4 groups of different curvatures. Finally, the third study^19^ used unsupervised learning to predict the risk of future keratoplasty based on corneal OCT parameters. Their model separated the patients in non-overlapping clusters of different keratoconus severity and other corneal conditions, from which they calculated the likelihood of future keratoplasty.

Regarding methodology, we used the central 50 × 50 values of each matrix because of a large number of missing values in the peripheral data which could affect clustering accuracy. Before using the dimensional reduction algorithm we selected, it is usually recommended to reduce the number of dimensions to around 50 dimensions with another dimensionality reduction algorithm such as Principal Component Analysis. We found that the algorithm was efficient even using the raw 10,000 dimensions. Thus, to simplify the methodology and to use the totality of the raw data, we decided not to add another dimensionality reduction technique. This was possible, probably because of the highly correlated nature of the raw topography data. It should be noted that corneal topography data is highly standardized, and typical examinations of each clustered diagnosis are very similar to each other (see Supplementary Figure S1). This explains why flattening the data to a 10,000 dimensions vector did not impair the clustering abilities of the algorithm. Considering the nature of the data, a convolutional neural network could have been used as a feature extractor before performing clustering. However, most pre-trained networks available use 3 input channels instead of 4 as we would have needed here.

We acknowledge that it is usually preferred to have the data be annotated manually by 3 different experts. Although only 2 experts performed the labeling in this study, our goal was not to match human diagnostic capabilities but to roughly sort and extract examinations from a noisy dataset.

Studying the dimensionality reduction algorithm results, it is interesting to note that most right eyes have been correctly separated from most left eyes (Figs. 1 and 2, Supplementary Video 1 and 2). For RS patients on the other hand, this separation was not as obvious. This is probably due to the fact that the laser treatment tends to erase natural corneal asymmetry in its central part. It should be noted that the 3 created parameters (x, y and z) do not represent any known parameter and would be different for every run of the algorithm and every dataset. Therefore they do not help understand how the algorithm separated the data and do not allow the comparison of the results to another dataset, but only offer a 3 dimensional representation of the dataset, and the subsequent use of a clustering algorithm which would be inefficient on a 10,000 dimensions dataset.

The selected clustering algorithm is useful in real unclean datasets containing noise, such as this one. Indeed, we found that excluded examinations had more missing values on average and comprised most examinations manually labeled as ‘Other’. It also allows the detection of complex structures in data. We can see this through the shape of the ‘Normal’ cluster, which has a hole in its center. This suggests that the algorithm correctly extracted the underlying complex structure of the core “Normal” cluster. Although no “Fuchs” cluster was detected by the algorithm, it is interesting to note that most “Fuchs” examinations were grouped in the same area of space in the dimensionality reduction process (Fig. 1, Supplementary Video 1, supplementary Fig. S1, green datapoints). Therefore one could suppose that more examinations with a diagnosis of Fuchs corneal dystrophy would have created a denser cluster in that area that could have been detected by the clustering algorithm. It should be noted that the “Other” diagnosis class is heterogeneous. It is composed of low quality examinations with many missing values, artefacts and examinations from less frequent diagnoses such as pterygium, scars of infectious keratitis and patients who received penetrating keratoplasty, or radial keratotomies. These are data that are not similar to each other and therefore have no reason to be located together in space and constitute a cluster.

This methodology might not be directly applicable to other datasets. Indeed, the dimensionality reduction algorithm parameters depend on the dataset itself. Although the provided parameters can be a starting point when using a similar dataset, they might need some tuning for optimized results on another dataset. This is a potential limitation, as several hours of computational time is needed for each run of the algorithm. Also, this computational time increases non linearly with the number of examinations, and thus it could be difficult to find optimal parameters on bigger datasets.

As we found that the number of missing values per examination was related to classification error rate, it could be interesting to first exclude the examinations having a number of missing values above a specific threshold to achieve better accuracy. But this would imply analyzing the missing values distribution beforehand, and assigning a somewhat arbitrary value to that threshold. Using the clustering algorithm capabilities to exclude examinations that are too different from the clusters examination is a simpler process that can be used even with little or no expert knowledge on the type of data used and is therefore applicable to any kind of numerical data.

In summary, we have shown that unsupervised techniques can be used efficiently to ease and automate the processes of constituting large groups of corneal topography examinations for research purposes. Higher error rates compared to diagnostic or screening tests are acceptable in this context. These methods could be used on any other type of digital examinations of which we have large databases, to rapidly sort the cases in groups of the most common diagnoses to be used subsequently in more powerful studies with a great number of observations. This process could be facilitated by manufacturers by allowing exportation of all examinations’ raw data contained in the devices at once.

## Method

### Data collection

**Figure 3:**
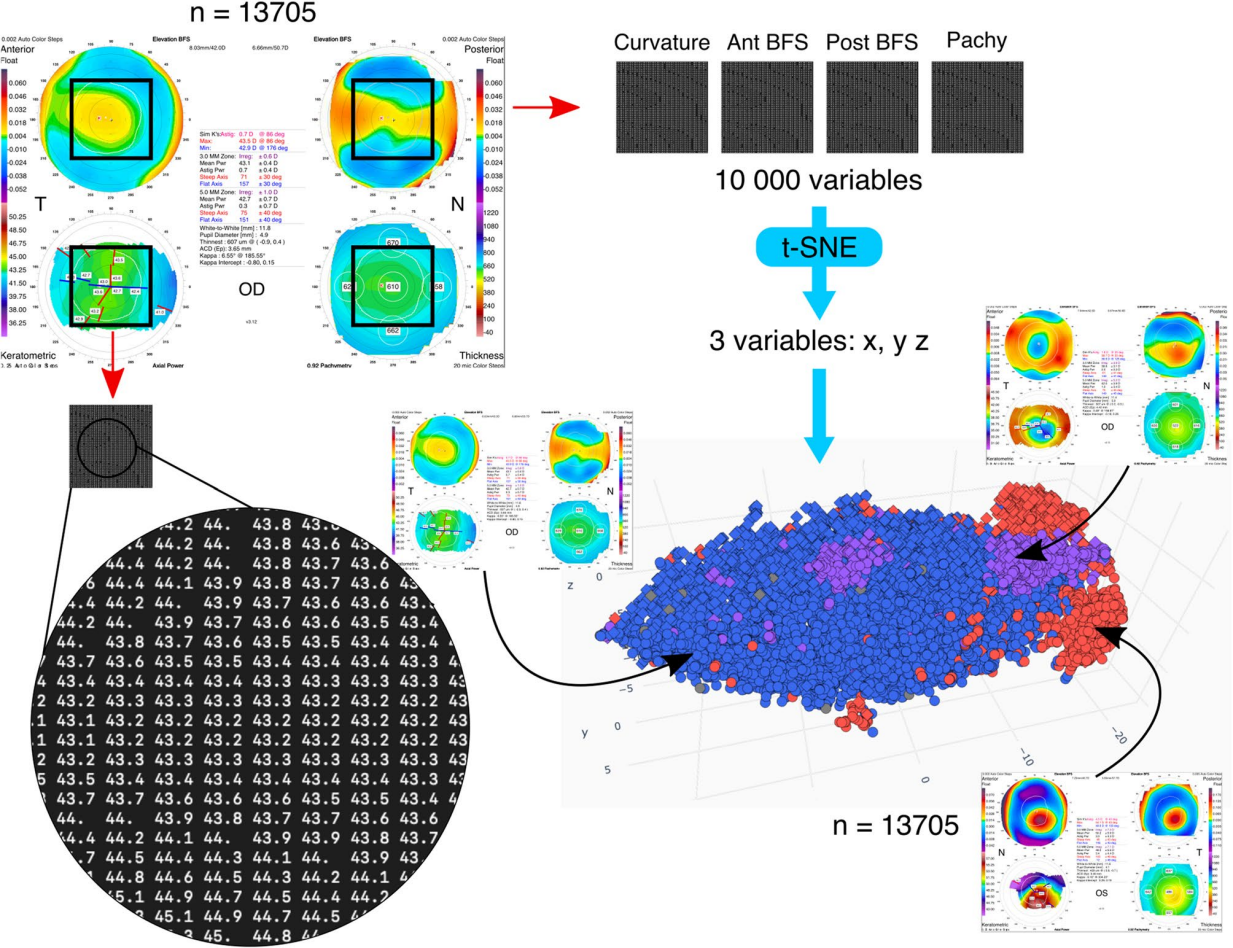
Schematic representation of the methodology. Values of 4 different corneal topography maps are used: ‘Elevation against the anterior BFS’, ‘Elevation against the posterior BFS’, ‘Axial anterior curvature’, and ‘Pachymetry’. Each map is a matrix composed of 50 × 50 elements. An enlargement of the anterior axial curvature matrix is shown in the lower left corner. All data points can be visualized in 3 dimensions after dimensionality reduction by t-SNE algorithm (bottom right). Examinations are roughly grouped by diagnosis. t-SNE = t-distributed Stochastic Neighbor Embedding, Curvature = Anterior axial curvature matrix, Ant BFS = Elevation against the anterior Best Fit Sphere matrix, Post BFS = Elevation against the posterior Best Fit Sphere matrix, Pachy = Pachymetry matrix.

This study was approved by the Institutional Review Board at Rothschild foundation and followed the tenets of the Declaration of Helsinki. Informed consent was obtained from all subjects. 22,066 Orbscan (Bausch&Lomb, U.S.A) examinations were randomly extracted from our Orbscan database using the batch export functionality. The last examination of the first visit for each eye of each patient was selected. This process reduced the number of examinations to 13,705. The raw data from 4 commonly used maps in clinical practice was selected for each examination: ‘Elevation against the anterior Best Fit Sphere (BFS)’,´Elevation against the posterior BFS’, ‘Axial anterior curvature’, and ‘Pachymetry’. Each map was a square matrix of 100 × 100 numerical values. Each matrix was cropped to its 50 × 50 central elements. The 4 maps thus constituted a total of 4 × 2500 = 10,000 numerical values (Fig. 3).

### Tracers and manual labeling

A sample of 248 examinations were randomly selected, and will be referred to as “Tracers” in the rest of this article. All tracers were manually reviewed and labeled. The 4 most represented identifiable diagnoses were used as classes for the clustering experiment of the whole dataset. Those classes were, “Normal”, “Keratoconus (KC)”, “History of Myopic Refractive surgery (RS)”, “Fuchs corneal dystrophy (Fuchs)”. A fifth class named “Other” was constituted of all examinations that could not be assigned to a specific class with a good level of confidence, including bad quality examinations. All 13,705 examinations were manually labeled and checked by two corneal topography experts (5 years of practice in a corneal and refractive surgery department) in a random order. Examinations were classified as “KC” if the anterior curvature map showed one of the classic keratoconus patterns described by Rabinowitz et al.^20^ associated with corneal thinning. “RS” examinations were cases that underwent myopic laser surgery and had an oblate anterior surface (flat in its center), a prolate posterior surface (steep in its center) , a central corneal thinning and lower central curvature values compared to the periphery. Finally, “Fuchs” examinations had a central pachymetry over 600 microns with reduced peripheral thickness in the corneal periphery and an oblate posterior surface. This pattern suggests a Fuchs corneal dystrophy with central corneal edema. Our goal was not to achieve state-of-the-art diagnostic capabilities but merely to see if an unsupervised learning algorithm could help facilitate the extraction and sorting of a usable fraction of the dataset, as a human would do by simply reading the examinations. Therefore, all manual labeling was performed by solely analyzing the examinations, with no knowledge of the patients’ medical records.

**Figure 4:**
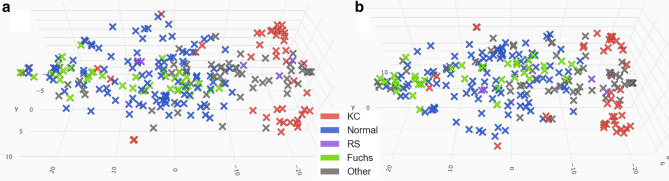
Three-dimensional representation of the Tracers examinations’ locations after dimensionality reduction (**a**: Top view, **b**: Bottom view). Unlabeled data has been removed. Ground truth diagnoses are color coded.

“Tracers” examinations were used together with the unlabelled data to quickly assess the relevance of the dimensionality reduction and clustering algorithm, and choose the best parameters (Fig. 4). Indeed, labeled examinations of the same class should be roughly grouped together and separated from examinations of other classes. Metaphorically, they can be compared to radioactive tracers in the human body from which we can suppose the location of non-radioactive similar molecules (in our case, unlabeled similar data).

### Data preprocessing and algorithms

Minimal data preprocessing included 3 steps: missing value replacement, scaling and dimensionality reduction. Missing values were replaced from all maps by the mean value for each matrix element, which is the simplest data replacement strategy. Data from each type of map were scaled between 0 and 1 using data from all patients^21^. Finally, dimensionality reduction from 10,000 to 3 dimensions was performed using the t-SNE algorithm^22^ (t-distributed Stochastic Neighbor Embedding) (see Table 3 : Glossary) with a perplexity of 50, a learning rate of 1000 and an angle of 0.9 (Figs. 1 and 3). Clustering was performed on all 13,705 patients with HDBSCAN algorithm^23^ using values of 53 and 325 for minimum samples and minimum cluster size parameters respectively. (Clustering results obtained from other parameter combinations are available in supplementary Figure S3).

**Table 3:** Glossary.

Terms	Description
HDBSCAN (Hierarchical Density-Based Spatial Clustering of Applications with Noise)	HDBSCAN automatically clusters examinations based on the density of data points in space and the distance between points and dense areas. It has the ability to exclude certain examinations from the clustering if they are considered as noise
Minimum cluster size	Minimum cluster size is a HDBSCAN parameter which controls the minimum number of points in a cluster
Minimum samples	Minimum samples is a HDBSCAN parameter which controls the initial dilation of space: points in less dense areas are viewed even further away from the other points
t-SNE (t-distributed Stochastic Neighbor Embedding)	t-SNE is a non-linear dimensionality reduction algorithm. The t-SNE algorithm builds a 2 or 3 dimensional representation of the data in which points that are close to each other in high dimensional space are also close in this low dimensional space
Angle	Angle is a t-SNE parameter used to speed up calculation by considering points very close to each other as only one point (values closer to 1 implies more approximation)
Gradient descent	Gradient descent is a very commonly used algorithm to iteratively optimize a complex function. In t-SNE, it is used to minimize the difference between the distributions of pairwise distances of points in high dimension and in low dimension
Learning rate	Learning rate is a t-SNE parameter which controls the updates of the gradient descent algorithm (see gradient descent)
Perplexity	Perplexity is a t-SNE parameter which controls the balance between local and global structure in the data. A smaller value tends to create many small clusters while a larger value tends to create fewer larger clusters

### Performance indices and statistics

For each constituted cluster, the number of examinations of each ground truth diagnosis was counted. The diagnosis class of each cluster was assigned to the most represented diagnosis of each cluster. Performance of the clustering process was evaluated through overall accuracy, and diagnosis-wise, sensitivity and specificity. Overall accuracy was defined as the number of correctly labeled observations divided by the total number of observations included by the clustering algorithm. All performance measurements were calculated on both tracers and unlabeled data, as they were considered equally by the algorithm and might as well be erroneously labeled.

The number of missing values of each examination (before missing values replacement) was counted. To test the relationship between the number of missing values and a correct or incorrect classification by the algorithm, we used the Chi Square independence test. The examinations were grouped in two categories: less than 1% and more than 1% of missing values per examination. The mean and standard deviation of missing values in correctly and incorrectly classified labels were compared as well as in examinations included and excluded by the clustering algorithm with a Student t test after testing for normality of data distribution with Kolmogorov–Smirnov test. *P*-value < 0.05 were considered significant.

### Programming language and libraries

All calculations, algorithms and figures were done in python 3.6. Scikit-learn library’s algorithms was used for t-SNE. HDBSCAN library was used for clustering.

Seaborn and Plotly libraries were used for figure plotting. Statistics were calculated using Scipy Stats module.

## Supplementary information

Supplementary file1

Supplementary file2

Supplementary file3

## Data Availability

The datasets generated during and analysed during the current study are available from the corresponding author on reasonable request.
