# Supplementary material for: Unsupervised learning for large-scale corneal topography clustering
Source: Sci Rep. 2020 Oct 12;10:16973. doi: 10.1038/s41598-020-73902-7 (PMC7550569; doi:10.1038/s41598-020-73902-7)
Supplement: Supplementary file 1 — Supplementary file1 [file 41598_2020_73902_MOESM1_ESM.pdf]

Unsupervised learning for large-scale corneal topography clustering.

Pierre Zéboulon[1] MD, Guillaume Debellemanière[1], MD, Damien Gatinel[1,2] MD, PhD *

(1) Department of Ophthalmology, Rothschild Foundation, 25 Rue Manin, 75019 Paris, France

(2) CEROC (Center of Expertise and Research in Optics for Clinicians) Paris, France

SUPPLEMENTARY MATERIAL

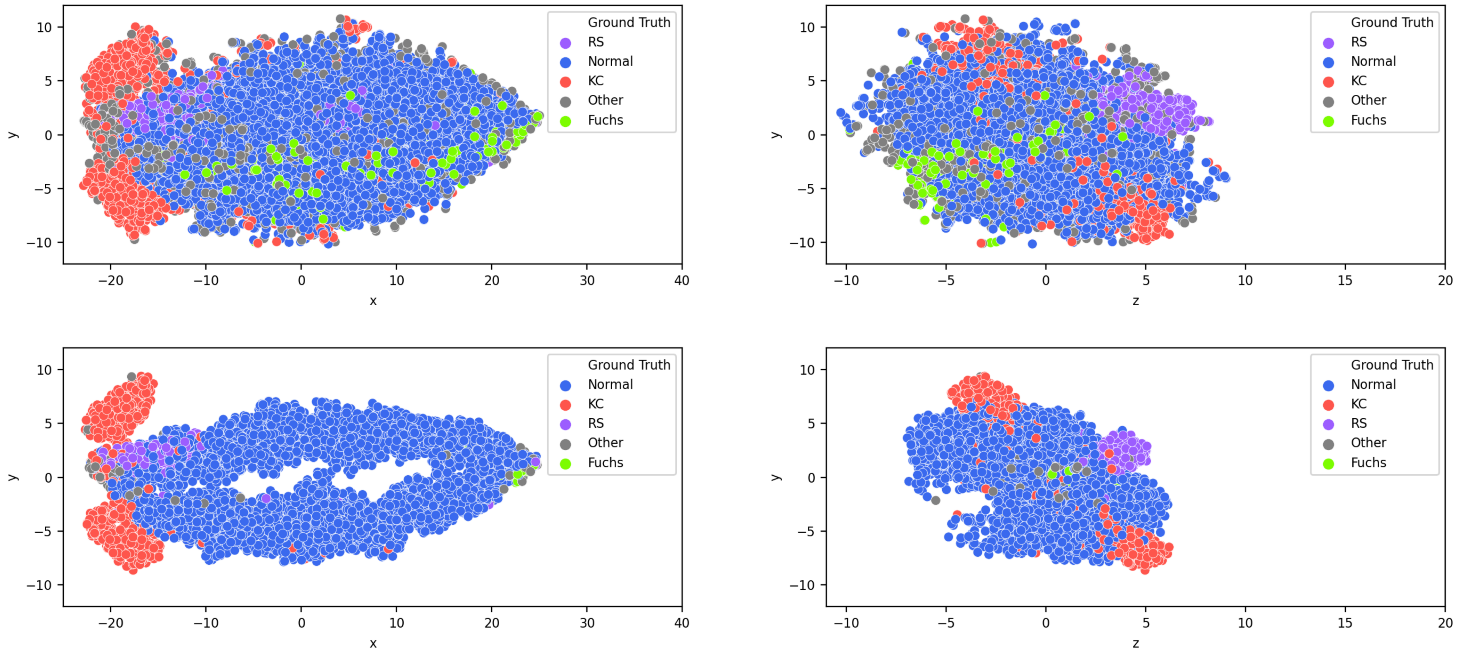

Supplementary Figure S1: 2D representations of the dataset (top row) and clustering algorithm results (bottom row
Points are colored by ground truth labels.
Each plot uses 2 components (x and y) or (z and y) of the t-SNE results.

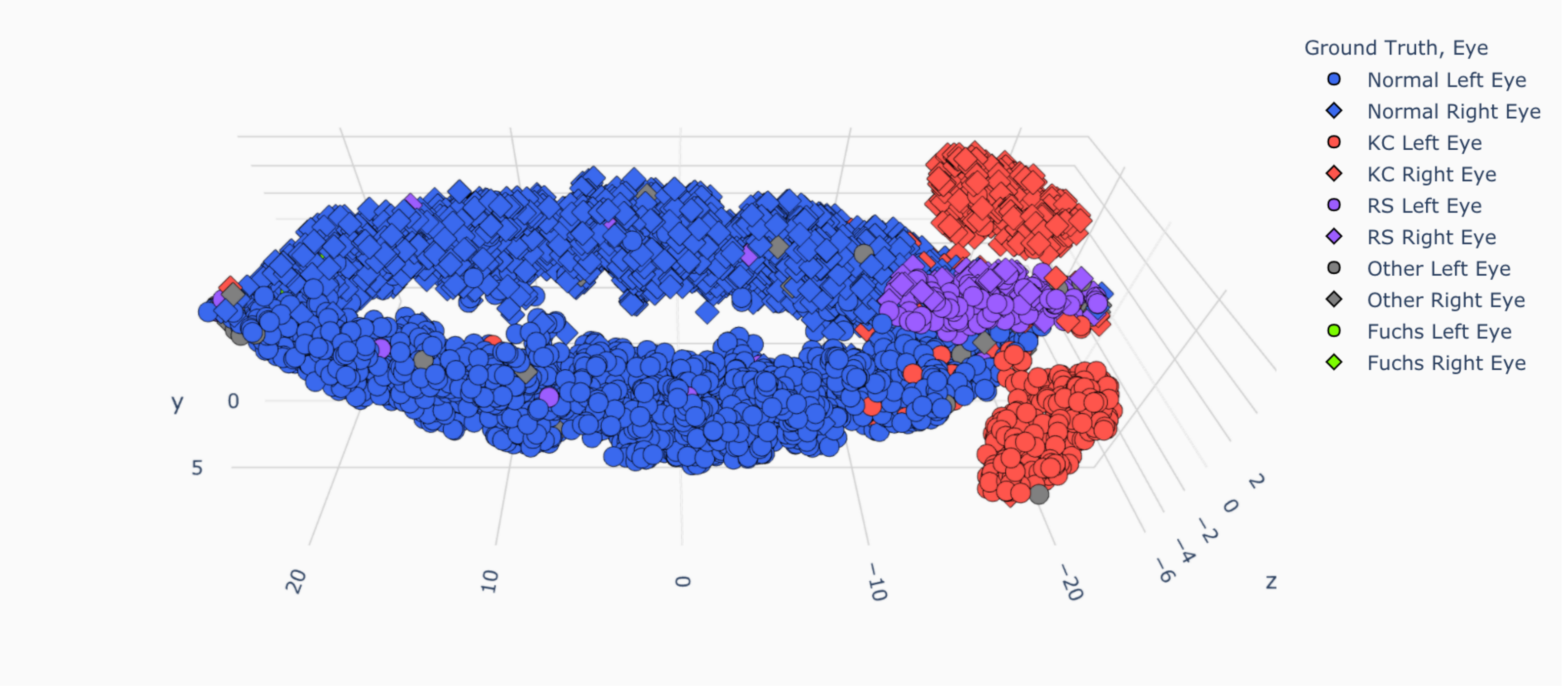

Supplementary Figure S2: 3D representation of the clustering results.
Points are colored by ground truth labels. Points that could not be clustered by the algorithm are not shown.

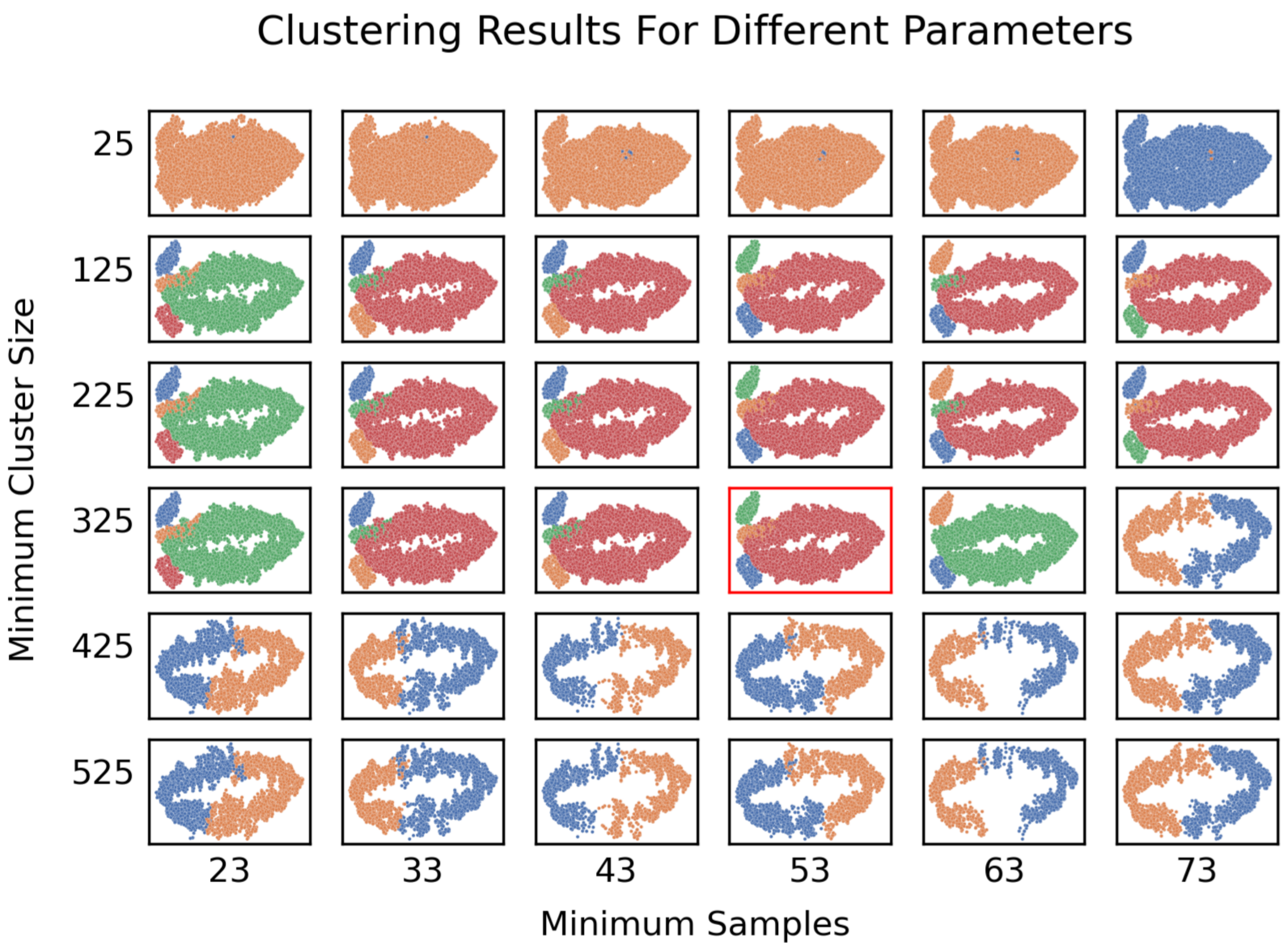

Supplementary Figure S3: 2D representations of the clustering algorithm results, for different combinations of the two hyper-parameters Minimum Samples and Minimum Cluster Size.
The combination used in the article is highlighted with red borders.
Points are colored by cluster for each plot.
The choice of colors is arbitrary and depends on the order of cluster composition by the algorithm.
Points excluded by the algorithm are not shown.
Clustering was performed on the 3 dimensional t-SNE results.
Each result is represented only by its x and y components.

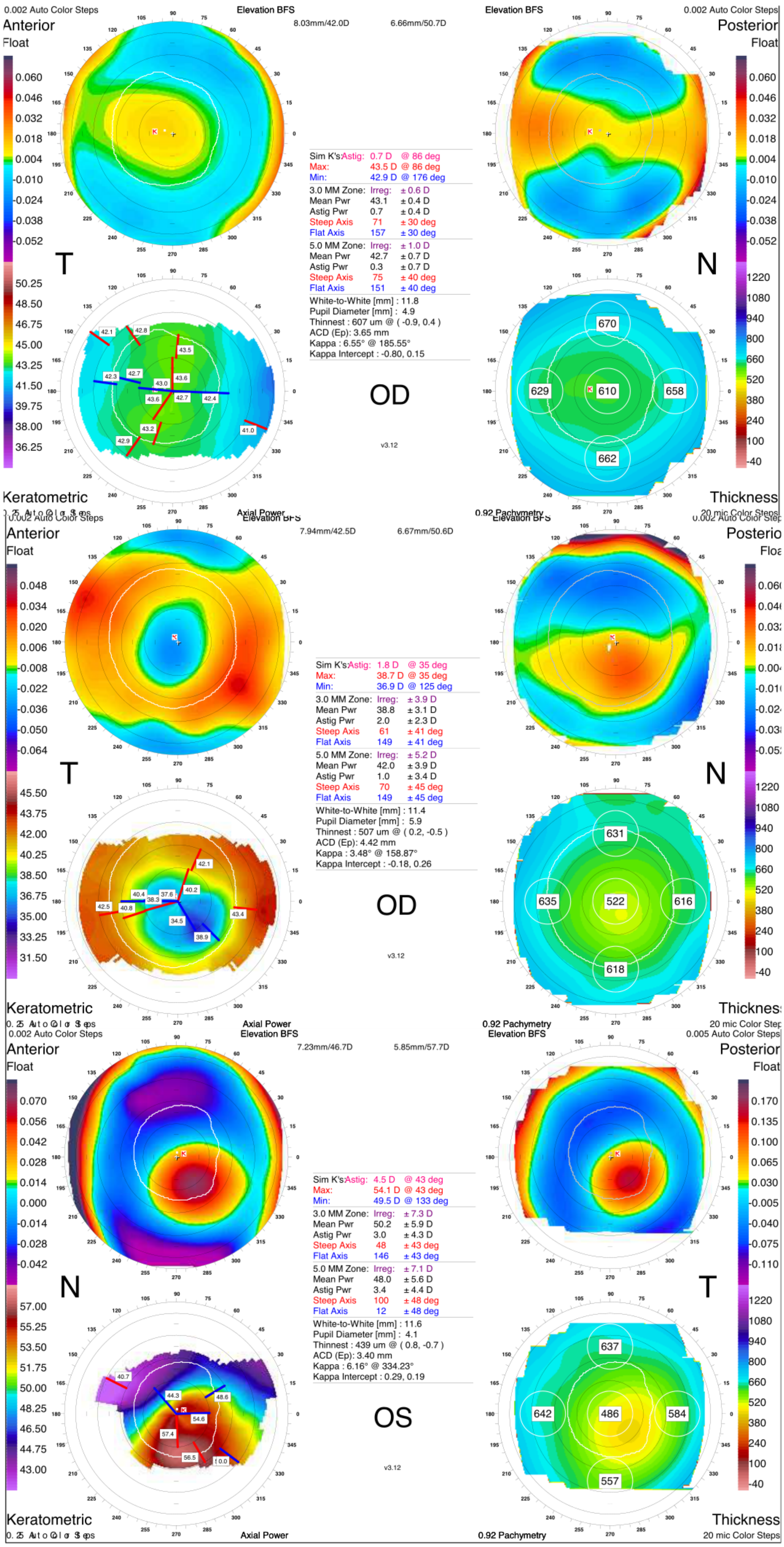

Supplementary Figure S4: Typical examinations for the 'Normal' (top), 'RS' (middle) and 'KC' (bottom) classes
